# Associations Among Changes in Body Composition, Activity, Muscle Recovery, and Obesity Following Total Knee Arthroplasty: A Retrospective Study

**DOI:** 10.7759/cureus.72282

**Published:** 2024-10-24

**Authors:** Kyohei Nagayama, Takaaki Shishido, Tsunehito Ishida, Norihiko Iwasa, Yohei Nishikawa, Toshiyuki Tateiwa, Toshinori Masaoka, Kengo Yamamoto

**Affiliations:** 1 Orthopedics, Tokyo Medical University Hospital, Tokyo, JPN; 2 Orthopedic Surgery, Tokyo Medical University Hospital, Tokyo, JPN

**Keywords:** bmi, body composition, body mass index, obesity, rehabilitation, total knee arthroplasty

## Abstract

Purpose: Total knee arthroplasty (TKA) is expected to improve knee pain and enable patients to regain the ability to walk, but the associations between preoperative and postoperative changes in body composition, muscle strength, and activity remain unclear. In this study, we investigated the association between changes in body composition before and after TKA surgery, the recovery process of muscle strength, and changes in activity, as well as the effects of obesity on TKA.

Methods: A total of 124 patients with unilateral knee osteoarthritis (OA) who underwent TKA were retrospectively evaluated. Body weight (BW) and body composition (body fat percentage (BFP) and diseased limb muscle mass (DLMM)), measured by bioelectrical impedance analysis, quadriceps muscle strength (QMS), measured using the isometric muscle strength meter, number of steps (NOS), and Japanese Orthopaedic Association (JOA) OA knee diseases treatment outcome criteria (JOA score) before the operation, at postoperative six months (PO6M), and at postoperative one year (PO1Y) after the operation were investigated and compared between the non-obese (BMI < 25 kg/m^2^) group, including underweight (<18.5 kg/m^2^) and normal weight patients (18.5 to 24.9 kg/m^2^), and obese (BMI ≥ 25 kg/m^2^) group, including overweight (25.0 to 29.9 kg/m^2^) or obese patients (≥30.0 kg/m^2^).

Results: In all patients, JOA scores significantly improved from 59.7 preoperatively to 81.2 at PO6M, and 82.7 at PO1Y (both p < 0.01). QMS significantly increased from 112.4 N preoperatively to 144.0 N at PO6M, and 151.0 N at PO1Y (both p < 0.01). On the other hand, there was no significant change in BW, BFP, NOS, and DLMM. A comparison between the obese (n = 76) and non-obese (n = 48) groups demonstrated that there was no significant difference in JOA scores preoperatively, but scores at PO1Y were significantly higher in the non-obese group (p < 0.01), and QMS increased significantly postoperatively in both groups, but the enlargement rate at PO1Y was 1.4% in the obese group versus 10.4% in the non-obese group (p = 0.04).

Conclusion: In the body composition of TKA patients, BW, BFP, and DLMM/BW did not change significantly from preoperatively to PO1Y, but QMS and JOA scores showed significant improvement at PO6M and PO1Y compared to preoperatively. The obese patients showed less improvement in JOA scores than non-obese patients, and the rate of increase in QMS at PO1Y was smaller, suggesting that rehabilitation focusing on muscle-strengthening training is necessary even at PO6M, especially in obese patients.

## Introduction

With the rapid aging of society in recent years, the number of patients with knee osteoarthritis (OA), a degenerative joint disease associated with aging, has been increasing substantially. Total knee arthroplasty (TKA) surgery is widely performed for end-stage OA [1,2] and is expected to continue to increase in the future. Although TKA surgery is considered a beneficial procedure as it is expected to improve patients’ pain and regain walking ability, the association between preoperative and postoperative changes in body composition, muscle strength, and activity remains unclear. Obesity is a risk factor for the development and progression of OA, and an increase in body mass index (BMI) of 5 kg/m2 is said to almost double the risk of OA progressing to the point of requiring TKA [3] but the effects of obesity on the clinical outcomes of TKA remain unclear to date.

Therefore, the aims of this study were (1) to clarify the relationship between changes in body composition and the recovery process of muscle strength and changes in activity level before and after TKA surgery using the bioelectrical impedance analysis (BIA) method, and (2) to examine the impacts of obesity on TKA. Investigation of these relationships is expected to help improve postoperative therapy to improve clinical outcomes. Furthermore, it may provide new insights into the need to modify rehabilitation programs based on the presence or absence of obesity. While BIA is a tool that can easily evaluate body composition in a clinical setting without physical invasion, it is not yet widely used in the orthopedic surgery field, and there are few reports quantifying body composition changes before and after TKA in particular [4].

## Materials and methods

Patients

This study was approved by the Institutional Review Board of Tokyo Medical University (study approval number: T2022-0067). A total of 124 patients with unilateral knee OA in 124 joints (104 joints of 104 women and 20 joints of 20 men) who underwent TKA surgery at our institution between July 2018 and March 2020, and who underwent follow-up examinations for at least one year after surgery were included in the analysis. Indications for TKA were severe knee pain and functional loss unresolved with nonoperative treatment modalities, and radiographically progressive OA changes with Kellgren-Lawrence (KL) grade III or IV [5]. Unilateral knee OA was defined as contralateral KL grade Ⅱ or less with no painful disability. Patients who were followed for less than one year, and those with rheumatoid arthritis, hemophilic arthritis, traumatic arthritis, and femoral condylar necrosis were excluded from the analysis.

Surgery

All surgeries were performed under general anesthesia by five senior surgeons (KY, TS, TM, TT, and TI). The cruciate retaining (CR)-type Stryker Scorpio Knee system (Stryker Orthopaedics, Mahwah, NJ) was used in all surgeries. Preoperative planning was performed by the mechanical alignment method. Intraoperatively, the midvastus approach was used, osteotomy was performed using the measured resection technique, and the patella was replaced in all patients. All implants were cemented.

Rehabilitation

The rehabilitation protocol for TKA was created by several orthopedic surgeons and rehabilitation physicians. Muscle training focusing on the quadriceps, gait training, and range of motion training using continuous passive motion (CPM) were started on the day after surgery. The goal of gait training was to walk up and down the stairs with a cane, and patients were discharged from the hospital approximately three weeks after surgery (average: 22.2 ± 5.4 days). After discharge from the hospital, support including that from physiotherapists is provided for a short period (up to six months after surgery), such as detailed information on self-rehabilitation using pamphlets, and continuation of outpatient rehabilitation after discharge.

Evaluation of body composition, quadriceps muscle strength, and number of steps

Examinations were conducted three times, namely, preoperatively, postoperative six months (PO6M), and postoperative one year (PO1Y) after the operation. The items examined included body weight (BW) and body composition, quadriceps muscle strength (QMS), number of steps (NOS), and the Japanese Orthopaedic Association (JOA) OA knee disease treatment grading criteria (JOA score). BW and body composition were measured by BIA using inner scan 50 V (TANITA, Tokyo, Japan) to determine body fat percentage (BFP), and total body and affected limb muscle mass (diseased limb muscle mass, DLMM) were measured. The BIA measurement was taken in the morning, each time in a standing position. To avoid the effects of food and exercise, the measurement was taken on an empty stomach, and exercise before the measurement was prohibited. Muscle strength was measured using the isometric muscle meter (μTas F1, ANIMA Corporation, Chofu, Japan) in the sitting position, with the seat height adjusted so that the feet were not placed on the floor, with the lower leg at 90° hip flexion and 90° knee flexion, and with a muscle strength measurement pad placed on the front surface of the affected lower leg, 20 cm from the lower patella. Measurements were taken three times by the same examiner (NK), and the average value was calculated. The number of steps taken was measured using a pedometer (HJ205IT-W, OMRON, Kyoto, Japan). The patients wore the pedometer throughout the day and were asked to submit a daily step count on a form. The JOA score (100 points in total) was evaluated using the following four categories: I = pain and ability to walk (30 points); II = pain and ability to climb stairs (25 points); III = range of knee motion (35 points); and IV = knee swelling (10 points). In the present study, the BIA method was used to investigate changes in body composition before and after TKA (preoperatively, PO6M, and PO1Y) and analyze their association with QMS, NOS, and JOA scores.

Comparison between the obese group and the non-obese group

All patients were categorized into the obese group or the non-obese group to compare the trends of each parameter over time. Patients with a BMI of ≥25 kg/m^2^ were in the obese group, and those with a BMI of <25 kg/m^2^ were in the non-obese group. Based on the World Health Organization classification of BMI [6], the obese group included overweight (25.0 to 29.9 kg/m2) and obese (≥30.0 kg/m2) patients, and the non-obese group included underweight (<18.5 kg/m2) and normal weight (18.5 to 24.9 kg/m2) patients. Muscle mass was corrected for body weight (DLMM/BW (%)) to adjust for gender and body size differences.

Statistical analyses

All statistical analyses were performed using JMP 14 software (SAS Institute Inc., Cary, NC). Wilcoxon signed rank and Fisher’s exact tests were performed to evaluate statistical differences in the gender proportions of obese and non-obese patients. The association between QMS and DLMM was analyzed using the Spearman correlation coefficient. A p-value of less than 0.05 was considered to indicate a statistically significant difference between groups.

## Results

All cases

**Figure 1:**
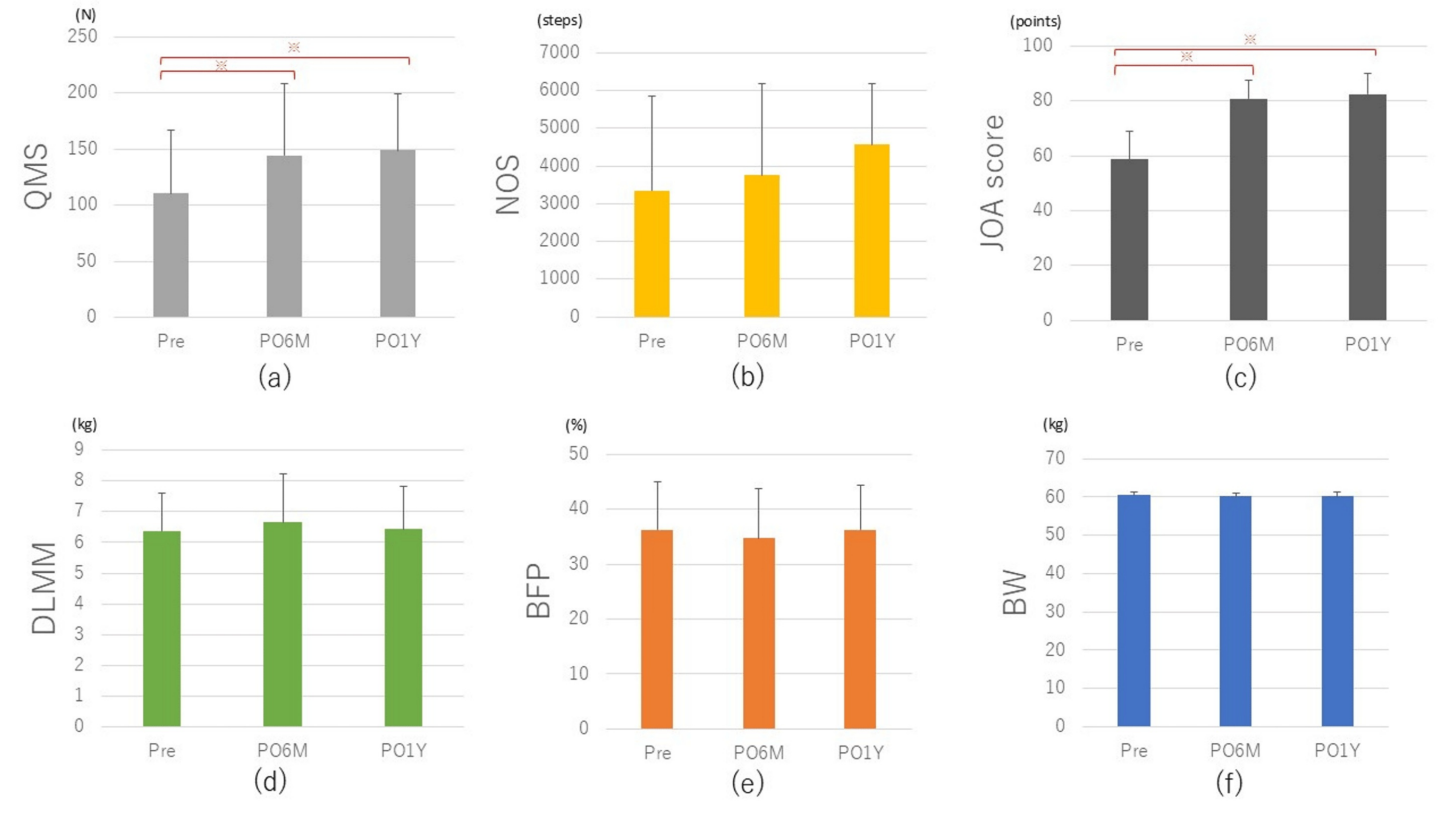
Graphs showing the changes in (a) QMS, (b) NOS, (c) JOA score, (d) DLMM, (e) BFP, and (f) BW at preoperative, six months postoperative, and one year postoperative period for all patients. * P < 0.01. QMS: quadriceps muscle strength; NOS: number of steps; JOA: Japanese Orthopaedic Association; DLMM: diseased limb muscle mass; BFP: body fat percentage; BW: body weight; Pre: preoperatively; PO6M: six months postoperatively; PO1Y: one year postoperatively.

The mean age (± standard deviation (SD)) at surgery was 75.2 ± 6.9 years, and the mean BMI was 26.1 ± 4.0 kg/m2. The temporal trends of each parameter in all patients (Table 1) showed that the JOA score increased significantly from 58.8 ± 10.2 points preoperatively to 80.8 ± 6.9 points at PO6M and 82.5 ± 7.5 points at PO1Y (both p < 0.01, Figure 1C). The QMS significantly increased from 112.4 ± 58.8 N preoperatively to 144.0 ± 64.0 N at PO6M and 151.0 ± 59.4 N at PO1Y (p < 0.01 and p < 0.01, respectively, Figure 1A). NOS was 3,278 ± 2,383 steps preoperatively and 3,537 ± 2,277 steps at PO6M, with little change, but 4163 ± 2486 steps at PO1Y, an increase compared to preoperatively and PO6M (p = 0.15 and p = 0.32, respectively, Figure 1B), with no significant difference. On the other hand, there were no obvious changes in BW, BFP, and DLMM before and after surgery (Figures 1D-1F). QMS and DLMM showed a correlation between these two parameters at all time points (preoperative: r = 0.34, p < 0.01; PO6M: r = 0.49, p < 0.01; PO1Y: r = 0.35, p < 0.01).

**Table 1:** Patient demographic and clinical characteristics. Values are indicated as mean ± standard deviation. Sex differences and left/right knee differences were tested by Fisher’s exact test. BMI: body mass index; JOA: Japanese Orthopaedic Association; Pre: preoperatively; PO6M: six months postoperatively; PO1Y: one year postoperatively.

Parameter	Total	Obese (BMI < 25)	Non-obese (BMI ≧ 25)	p-value
Gender: women/men	104/20	66/10	38/10	0.32
Age (years)	75.2 ± 6.9	75.1 ± 6.8	75.9 ± 6.5	0.66
Surgical side: right/left	64/60	38/38	26/22	0.71
Height (cm)	152.0 ± 6.9	151.2 ± 6.6	153.2 ± 7.2	0.10
Body weight (kg)	60.4 ± 10.5	65.4 ± 9.1	52.4 ± 7.2	0.01
BMI (kg/m^2^)	26.1 ± 4.0	28.5 ± 2.8	22.3 ± 2.1	0.01
JOA score (total)				
Preoperative	58.8 ± 10.2	56.8 ± 9.7	61.5 ± 10.5	0.08
PO6M	80.8 ± 6.9	78.9 ± 7.8	83.4 ± 4.4	0.04
PO1Y	82.5 ± 7.5	79.2 ± 6.9	86.8 ± 6.1	<0.01
Pain and ability to walk				
Preoperative	18.4 ± 5.4	17.3 ± 5.5	20.0 ± 4.9	0.05
PO6M	26.7 ± 2.9	26.1 ± 3.0	27.5 ± 2.6	0.15
PO1Y	27.1 ± 3.3	26.3 ± 3.3	28.0 ± 3.0	0.08
Pain and ability to climb stairs				
Preoperative	7.5 ± 4.7	6.1 ± 3.4	9.5 ± 5.6	<0.01
PO6M	18.3 ± 3.9	17.2 ± 4.5	20.0 ± 1.8	0.02
PO1Y	18.9 ± 4.3	17.1 ± 4.5	21.3 ± 2.8	<0.01
Range of motion				
Preoperative	26.5 ± 4.2	26.6 ± 4.1	26.3 ± 4.6	0.72
PO6M	26.3 ± 3.6	25.9 ± 3.3	26.9 ± 4.0	0.45
PO1Y	26.8 ± 3.6	26.0 ± 2.8	28.0 ± 4.1	<0.05
Swelling				
Preoperative	6.4 ± 3.2	6.8 ± 3.1	5.8 ± 3.4	0.28
PO6M	9.5 ± 1.5	9.8 ± 1.0	9.1 ± 2.0	0.15
PO1Y	9.7 ± 1.2	9.8 ± 1.0	9.5 ± 1.5	0.41

Obese group vs. non-obese group

**Figure 2:**
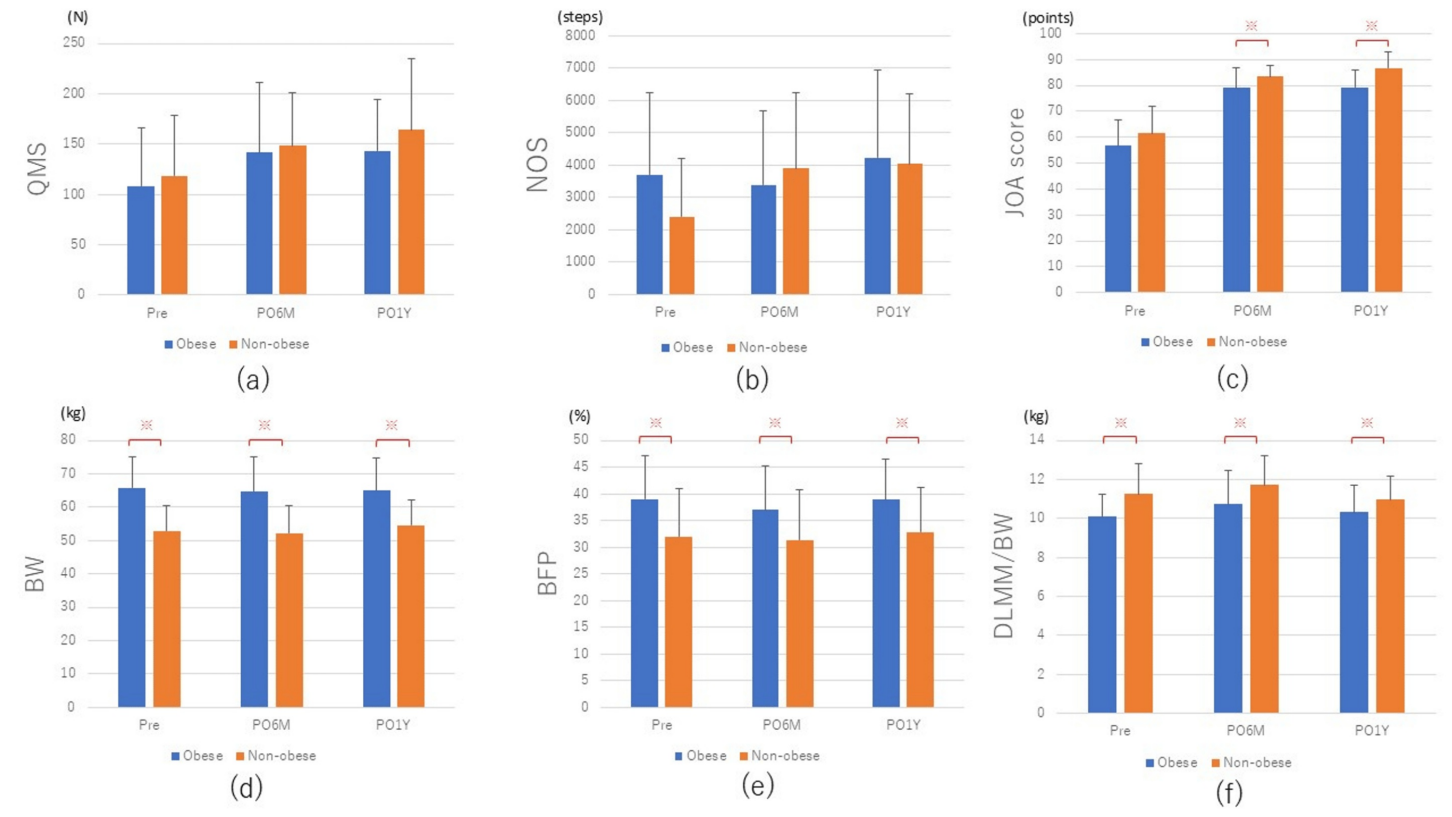
Graph showing comparisons between obese and non-obese patients. * P < 0.05. QMS: quadriceps muscle strength; NOS: number of steps; JOA: Japanese Orthopaedic Association; DLMM: diseased limb muscle mass; BFP: body fat percentage; BW: body weight.

**Figure 3:**
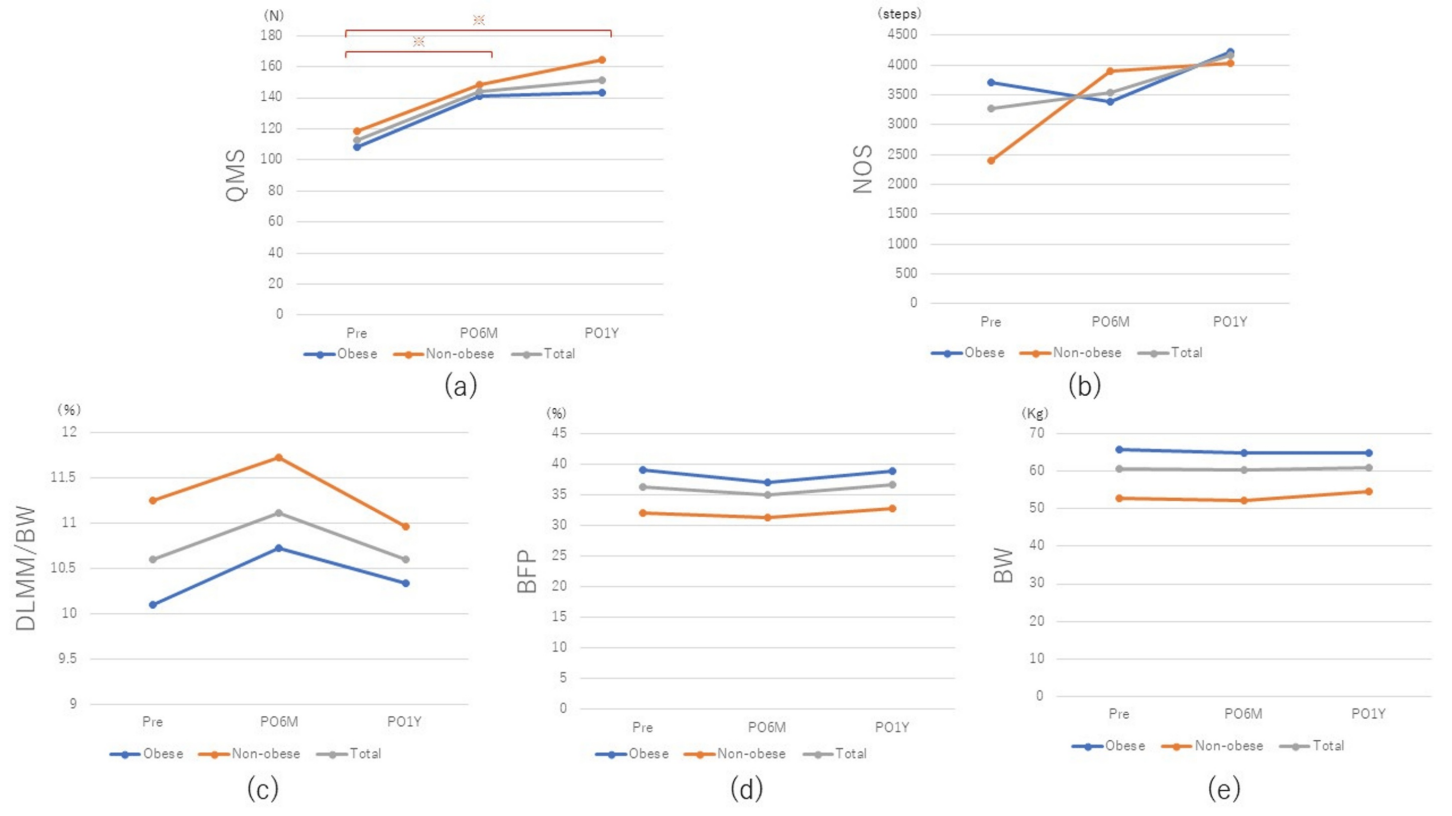
Graph showing the change in each parameter in obese and non-obese patients. * P < 0.05. QMS: quadriceps muscle strength; NOS: number of steps; DLMM: diseased limb muscle mass; BFP: body fat percentage; BW: body weight.

The obese group (mean: 28.5 kg/m2) with a BMI ≥ 25 consisted of 76 patients (10 men and 66 women), and the non-obese group (mean: 22.3 kg/m2) consisted of 48 patients (10 men and 38 women). There were no significant differences in mean age at surgery, sex ratio, affected knee (right/left), or height between the two groups (Table 1). QMS increased significantly in both the obese and non-obese groups (p < 0.01 and p = 0.03, respectively), from 108.6 ± 57.9 N and 118.6 ± 60.3 N, respectively, preoperatively to 141.4 ± 69.7 N and 148.6 ± 52.7 N, respectively, by PO6M (Table 2 and Figure 3A), but there was no clear significant difference between the two groups at each time point (Table 2). From PO6M to PO1Y, the non-obese group had an enlargement rate of 10.5% (from 149 N to 164 N) in QMS, while the obese group had an enlargement rate of 1.4% (from 141 N to 143 N) in QMS, indicating that the obese group had a significantly smaller enlargement rate after PO6M (p = 0.04). NOS tended to decrease slightly in the obese group, from 3,702 ± 2,540 steps preoperatively to 3,382 ± 2,282 steps at PO6M and increased to 4,232 ± 2,720 steps at PO1Y (Figure 2). On the other hand, in the non-obese group, there was an increase over time from 2,398 ± 1,798 steps preoperatively to 3,894 ± 2,345 steps at PO6M, and 4,039 ± 2,156 steps at PO1Y, although there was no clear significant difference at PO6M and PO1Y compared with preoperatively, there was an increasing trend (p = 0.10 and p = 0.08, respectively) (Table 2 and Figure 3B). BW did not change significantly in both the obese and non-obese groups, from 65.8 ± 9.4 kg and 52.9 ± 7.6 kg, respectively, preoperatively to 64.7 ± 10.3 kg and 52.2 ± 8.1 kg at PO6M, and at PO1Y, the non-obese group showed an increasing trend at 54.6 ± 7.7 kg, whereas the obese group showed no change, at 64.9 ± 9.7 kg (Table 2). DLMM/BW was significantly greater in the obese group compared to the non-obese group preoperatively and at PO6M and PO1Y (p < 0.01, p = 0.01, and p = 0.02, respectively, Table 2 and Figure 3).

**Table 2:** Changes over time of each parameter in obese and non-obese patients. Values are indicated as mean ± standard deviation. QMS: quadriceps muscle strength; NOS: number of steps; BW: body weight; BFP: body fat percentage; DLMM: diseased limb muscle mass; Pre: preoperatively; PO6M: six months postoperatively; PO1Y: one year postoperatively.

	Obese	Non-obese	p-value
QMS (N)			
Preoperative	108.6 ± 57.9	118.6 ± 60.3	0.24
PO6M	141.4 ± 69.7	148.6 ± 52.7	0.28
PO1Y	143.4±50.8	164.2 ± 70.7	0.12
NOS (steps)			
Preoperative	3,702 ± 2,540	2,398 ± 1,798	0.11
PO6M	3,382 ± 2,282	3,894 ± 2,345	0.57
PO1Y	4,232 ± 2,720	4,039 ± 2,156	0.98
BW (kg)			
Preoperative	65.8 ± 9.4	52.9 ± 7.6	<0.01
PO6M	64.7 ± 10.3	52.2 ± 8.1	<0.01
PO1Y	64.9 ± 9.7	54.6 ± 7.7	<0.01
BFP (%)			
Preoperative	39.0 ± 8.0	32.0 ± 8.9	<0.01
PO6M	37.0 ± 8.2	31.3 ± 9.4	<0.01
PO1Y	38.9 ± 7.7	32.8 ± 8.3	<0.01
DLMM/BW (%)			
Preoperative	10.1 ± 1.1	11.3 ± 1.6	<0.01
PO6M	10.7 ± 1.8	11.7 ± 1.5	0.01
PO1Y	10.3 ± 1.4	11.0 ± 1.2	0.02

## Discussion

Changes in body composition

Stets et al. reported a decrease in BW of more than 5% in 21.5% of patients at 20 months after TKA [7]. On the other hand, Zeni et al. reported that at two years postoperatively, an average BW increase of 6.4 kg was observed in 66% of the patients, together with an increase in muscle mass [8]. Thus, the results of weight change after TKA vary depending on reports, and there is no consensus. In the present study, there was no obvious increase or decrease in BW from the preoperative value at PO6M and PO1Y in our entire case series (Figure 1F). The obese group showed a slight decrease from preoperative (65.8 kg) to PO6M (64.7 kg) and no change from then to PO1Y (64.9 kg), whereas the non-obese group showed no change from preoperative (52.9 kg) to PO6M (52.2 kg) and then an increasing trend until PO1Y (54.6 kg) (Table 2 and Figure 3E). In the non-obese group, BW increased despite an increase in the number of postoperative steps and activity level, suggesting that weight fluctuation is more greatly affected by increased caloric intake owing to increased appetite than by changes in activity level [9]. BFP tended to decrease slightly at PO6M, and then increase slightly at PO1Y in all patients (Table 2). On the other hand, DLMM/BW was significantly higher in non-obese patients than in obese patients preoperatively (p < 0.01), and this trend remained unchanged until PO1Y. However, both obese and non-obese groups showed an increasing trend of DLMM/BW up to PO6M, followed by a decreasing trend from PO6M to PO1Y (Table 2 and Figure 3C).

Changes in muscle strength and activity

In this study, QMS in the obese and non-obese groups increased significantly at PO6M (p < 0.01 and p < 0.01, respectively) and PO1Y (p < 0.01 and p < 0.03, respectively) compared to preoperatively (Figure 3A). In addition, when the correlation between QMS and DLMM was analyzed, a weak correlation was observed for all patients at all observation periods. Therefore, it was considered that QMS could be speculated by measuring DLMM using the BIA method. Several studies have been reported on muscle strength changes after TKA surgery [10-13]. There are reports that the decrease in QMS continues for several years and that muscle strength recovered to only 70% of that of healthy subjects of the same age at PO1Y [11,12], suggesting that sufficient improvement in QMS cannot be expected in the natural course after TKA. On the other hand, a recent systematic review [13] using the parapatellar approach reported that QMS decreased the most at three days postoperatively, then began to increase after three months, increased to the same level as before the surgery at PO6M, and increased significantly at PO1Y compared with before surgery. In our present patients, a significant increase in muscle strength was observed at a relatively early stage (PO6M), indicating that muscle strength increased earlier than in previous reports of the parapatellar approach. This result may be associated with the fact that the midvastus approach performed at our department is less invasive on the knee extensor mechanism than the parapatellar approach, resulting in an earlier recovery of knee extensor strength [14]. In a study on the correlation between the rate of improvement in QMS after TKA and BMI, Wendelboe et al. found that QMS after TKA was not affected by BMI [15], whereas Silva et al. reported that QMS more than two years after TKA was weaker in patients with a higher BMI than those with a lower BMI [16]. Regarding our cases, QMS was significantly enhanced in both the obese and non-obese groups at PO6M, but from PO6M to PO1Y, the increase rate was 10.5% in the non-obese group and 1.4% in the obese group, indicating a small increase in the obese group from PO6M. Obesity is considered to affect the degree of symptomatic improvement after TKA, with a report [17] showing that the obese group had a significantly lower improvement rate (p = 0.05) than the non-obese group in terms of postoperative function of the Western Ontario and McMaster Universities Arthritis Index (WOMAC) score. However, there are also reports of similar postoperative TKA outcomes in obese and non-obese patients [18,19]. NOS and JOA scores were analyzed as indices of preoperative and postoperative activity, and the outcomes before and after TKA in this study. The JOA scores showed significant improvement at PO6M (p < 0.01) and PO1Y (p < 0.01), but the obese group had significantly lower scores at PO6M and PO1Y than the non-obese group (p = 0.01 and p < 0.01, Table 1). The obese group had significantly lower scores for pain and walking ability and pain and stair climbing than the non-obese group before surgery (Table 1), and in particular, the scores for pain and stair climbing were significantly lower at six months (p = 0.02) and one year (p < 0.01) after surgery, suggesting a significant effect on the total JOA score. The effect of obesity on the total JOA score was considered to be significant. Obesity is known to be a risk factor for prolonged postoperative pain and for the severity of postoperative pain [20]. There are many reports that corroborate the results of our present study, including a report that obesity (BMI) was negatively correlated with walking ability six months after TKA surgery [21] and that obese patients had lower functional outcomes, such as time up-and-go tests and delayed pain improvement [22]. Obesity increases the secretion of inflammatory cytokines and decreases the secretion of anti-inflammatory cytokines through hypertrophy and hyperplasia of adipose tissue [23], which may enhance inflammatory and nociceptive pain in the local area.

Application to rehabilitation and patient education

The results of our present study showed that QMS in both the obese and non-obese groups increased at PO6M, but between PO6M and PO1Y, the obese group showed a smaller increase in QMS than the non-obese group, suggesting the need for more rehabilitation intervention from PO6M, especially in the obese group. In DLMM/BW, both obese and non-obese groups showed an increasing trend up to PO6M and a decreasing trend from PO6M to PO1Y. This result also suggests that the no special intervention after PO6M may have affected the increase or decrease in DLMM/BW, and that rehabilitation intervention after PO6M needs to be considered not only for muscle strength but also for muscle mass volume. BW and BFP did not change before and after surgery. We had anticipated a reduction in body weight and body fat following TKA in obese patients, leading to improved postoperative pain and mobility. However, the results of this study did not demonstrate significant decreases in body weight or body fat following TKA. Analysis of JOA scores postoperatively revealed insufficient improvement in pain relief and mobility enhancement in the obese group compared to the non-obese group. Additionally, objective measurements of walking steps postoperatively showed limited improvement in the obese group, indicating that TKA alone may not be sufficient to improve activity levels and exercise. Therefore, to further enhance clinical outcomes in obese patients, it was considered essential to implement long-term rehabilitation interventions aimed at not only strengthening muscles but also improving activity levels, along with adequate dietary interventions focusing on nutritional guidance for weight control.

Limitations

There are several limitations to this study that should be considered. First, the duration and stage of OA varied from patient to patient, which may have affected the results of body composition, activity, and muscle mass. Second, although men and women were included in the study, there were large sex differences in changes in body composition, muscle strength, and activity, and it would hence be desirable to analyze the results separately for men and women. The study period was short (up to PO1Y), and it is expected that body composition and muscle mass will change further over a longer period of time, so a longer follow-up is necessary. Third, this study considered only strengthening exercises, which is a small component of TKA rehabilitation. Although muscle strength was assessed only in the quadriceps in this study, Bade et al. reported that not only quadriceps muscle but also hamstring and ankle dorsiflexor muscle weakness occurs after TKA [24], and a comprehensive rehabilitation program after TKA should include ankle joint muscle strengthening in addition to quadriceps and hamstring strengthening to optimize safety and function. Therefore, a comprehensive rehabilitation program after TKA should also include ankle joint strengthening in addition to quadriceps and hamstring strengthening.

## Conclusions

In the body composition in patients following unilateral TKA, there were no significant changes in BW, BFP, and DLMM/BW from preoperative to PO1Y, but QMS and JOA scores showed significant improvements at PO6M and PO1Y compared to preoperative scores. The obese patients showed less improvement in JOA score than non-obese patients, and the rate of increase in QMS at PO1Y was also significantly smaller, suggesting that obese patients need rehabilitation focusing on muscle strengthening training at PO6M.
